# Vertical Stacking of Atomic-Layer-Deposited Oxide Layers via a Fluorinated Graphene Transfer Technique

**DOI:** 10.1021/acsnano.5c04669

**Published:** 2025-06-13

**Authors:** Hyunjun Kim, Huije Ryu, Hyun Woo Jeong, Jiwoo Kim, Donghoon Moon, Sahngik Aaron Mun, Cheol Seong Hwang, Min Hyuk Park, Jangyup Son, Gwan-Hyoung Lee

**Affiliations:** † Department of Materials Science and Engineering, 26725 Seoul National University, Seoul 08826, Republic of Korea; ‡ Functional Composite Materials Research Center, 58975 Korea Institute of Science and Technology (KIST), Jeonbuk, 55324, Republic of Korea; § Department of JBNU-KIST Industry-Academia Convergence Research, Jeonbuk National University, Jeonju 54896, Republic of Korea; ∥ Nanoscience and Technology, KIST School, University of Science and Technology, Seoul 02792, Republic of Korea

**Keywords:** M3D integration, van der Waals interface, atomic layer deposition, oxide films, transfer technique, fluorinated graphene

## Abstract

Monolithic three-dimensional (M3D) integration of semiconductor devices offers a distinct advantage over two-dimensional size scaling by achieving higher connection densities between the device layers. Still, it presents several technological challenges, including the fabrication of upper layers, where lattice mismatch complicates the deposition of high-quality oxide layers directly onto prefabricated devices. Additionally, high-temperature postprocesses can lead to intermixing and degradation of underlying layers. Here, we demonstrate a fluorinated graphene (FG) transfer technique that enables the integration of atomic-layer-deposited oxide semiconductors and dielectrics, overcoming lattice mismatch and minimizing intermixing. The dipole interaction between fluorine and carbon in FG enables the deposition of ultraflat and high-quality oxide thin films via atomic layer deposition (ALD). Upon heating to 400 °C, the dissociation of fluorine atoms from graphene (Gr) facilitates detachment and transfer of the oxide films. Using the FG transfer method, we fabricated multiple stacks of oxide thin films with clean van der Waals interfaces, effectively preventing intermixing during the postannealing process. Furthermore, we fabricated top-gate field-effect transistors (FETs) with MoS_2_ and ZnO channels by stacking Al_2_O_3_ as gate dielectric film, achieving high device performance thanks to a high-quality interface. We also demonstrate the transfer of patterned ALD-grown oxide thin films on a large scale using selective deposition and detachment of oxide thin films on the patterned FG. Our findings suggest that the FG transfer technique is a promising approach for advancing M3D integration and addressing challenges related to thermal budget constraints in semiconductor fabrication.

## Graphical abstract

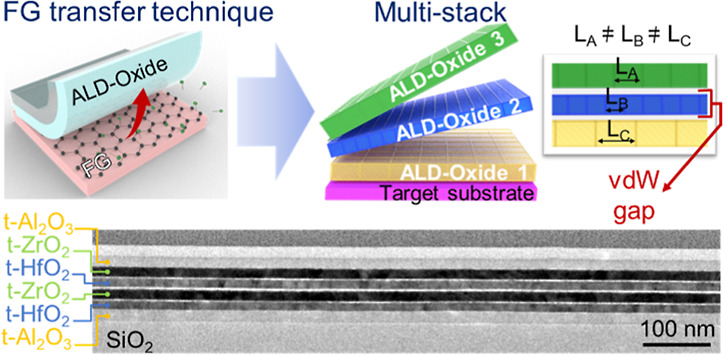

## Introduction

Monolithic three-dimensional (M3D) integration presents a promising approach to achieving high-density integration by vertically stacking components, thereby overcoming the two-dimensional scaling limitations in semiconductor technology.^1–10^ However, this integration technique faces several challenges during postprocesses, such as annealing and deposition, which can lead to material intermixing between the stacked layers, ultimately diminishing device performance.^11,12^ Additionally, when depositing materials on different substrates, lattice mismatch can degrade the crystalline quality and characteristics of the deposited material, further complicating the integration process.^13,14^ While buffer layers are commonly employed to mitigate intermixing and accommodate lattice mismatch, they often compromise key performance factors, such as capacitance density.^15,16^ Consequently, the upper layer in M3D integration is limited to using materials compatible with the underlying layers, and the fabrication processes should be carefully controlled to avoid introducing defects, strain, or degradation in the crystalline quality of the deposited materials.^11^ Here, we propose a fluorinated graphene (FG) transfer technique for integrating predeposited oxide layers. The dipole interaction between fluorine and carbon in FG enables ultraflat and high-quality deposition of oxide thin films via atomic layer deposition (ALD). By heating at 400 °C, fluorine dissociates from the graphene (Gr), allowing for easy detachment and transfer of the oxide films. The multiple stacks of oxide thin films fabricated by the FG transfer have clean van der Waals (vdW) interfaces, effectively preventing intermixing during postannealing. We also demonstrate the fabrication of high-performance top-gate field-effect transistors (FETs) with MoS_2_ and ZnO channels by transferring and placing the Al_2_O_3_ dielectric films on them as the gate insulator layer. Furthermore, patterned and in-inch-scale oxide thin films can be transferred, highlighting the high potential of the FG transfer technique in the M3D integration of ALD-grown oxide films.

## Results and Discussion

Figure 1a illustrates the FG transfer process of ALD-grown oxide films onto a target substrate. First, the Gr (exfoliated or CVD-grown) was placed on a SiO_2_ substrate, followed by fluorination by exposing it to XeF_2_ gas.^17–22^ Then, oxide thin films, such as Al_2_O_3_, HfO_2_, and ZnO, were grown on FG at 200 °C by ALD. The oxide/FG samples were heated on a hot plate at 400 °C for 4 h to break the C–F bonds, reducing the adhesion between the oxide films and Gr. Finally, the delaminated functional oxide films were transferred onto a target substrate by the pick-up technique (see Methods for details in the FG transfer process).^23^ Depending on how the polycarbonate (PC) film is applied to the target sample, the oxide film can be selectively picked up, either alone or together with the underlying Gr (as illustrated in Figure S1).

**Figure 1:**
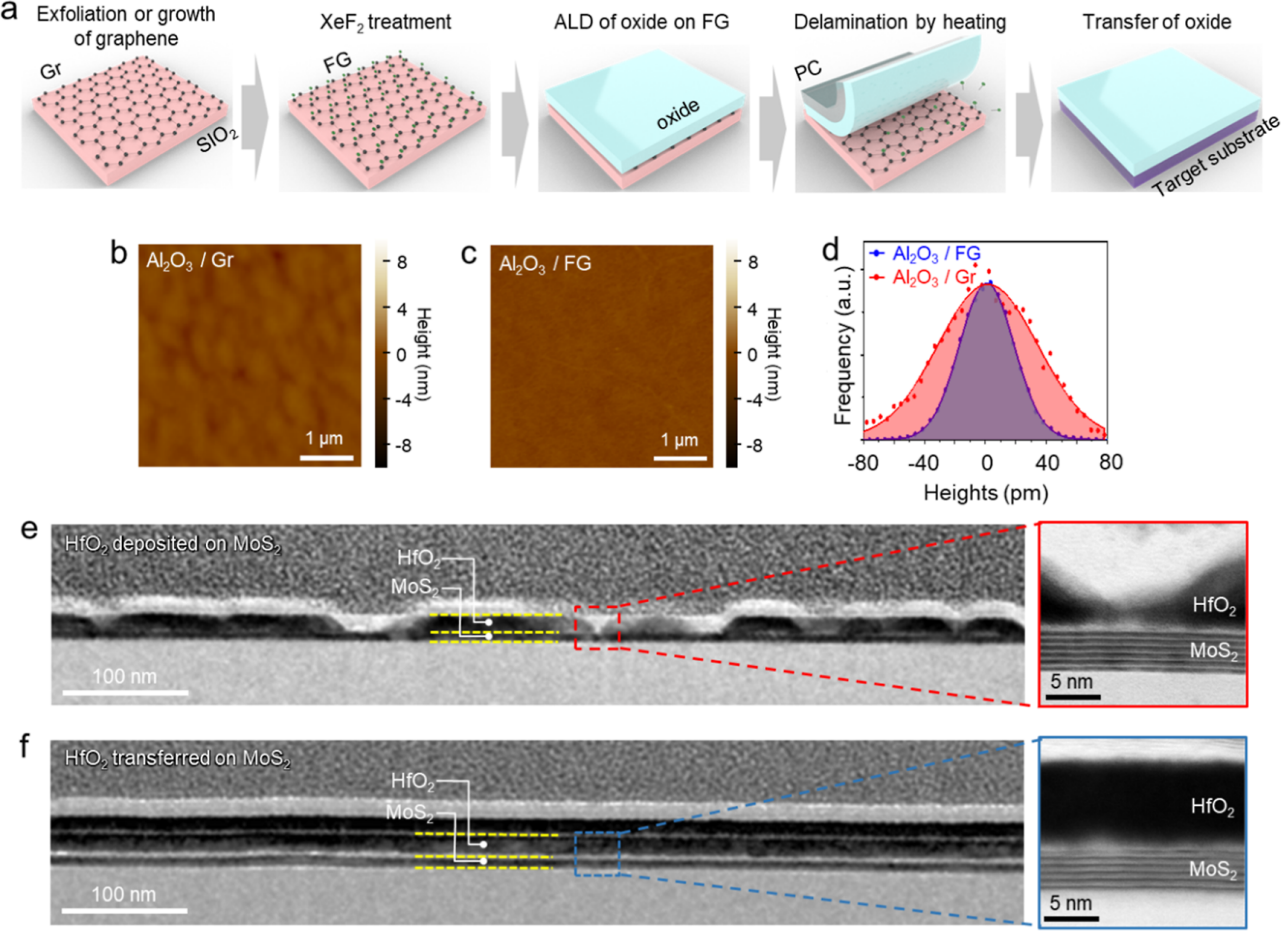
Fabrication of ALD-oxides by the FG transfer method. (a) Schematic of the FG transfer method. (b) AFM image of Al_2_O_3_/Gr. (c) AFM image of Al_2_O_3_/FG. (d) Surface roughness of the Al_2_O_3_/Gr and Al_2_O_3_/FG. (e) Cross-section TEM image of HfO_2_ deposited on MoS_2_. (f) Cross-section TEM image of HfO_2_ transferred on MoS_2_.

The atomic force microscopy (AFM) images of Figure 1b,c show that the Al_2_O_3_ film deposited on an exfoliated Gr at 200 °C by ALD exhibited an island-like morphology, consistent with previous reports that the absence of dangling bonds and nucleation sites makes it challenging to form a uniform oxide thin film on Gr.^16,24^ In contrast, a uniform and ultraflat Al_2_O_3_ film was deposited on the FG (see Methods for details of the ALD process and graphene fluorination). This improvement is attributed to the C–F dipoles that enhance the adhesion energy of H_2_O on FG, leading to increased coverage of the ALD-oxide thin film.^25–27^ Figure 1d shows that the surface roughness of the Al_2_O_3_ film, measured by AFM, deposited on FG (*R*_q_ = 0.171 nm) is much smaller than that on Gr (*R*_q_ = 0.392 nm).

HfO_2_ film on MoS_2_ is chosen to show the feasibility of the FG transfer process for improving the functional oxide/2D vdW channel material. For this demonstration, we prepared two samples: HfO_2_ was directly deposited on MoS_2_ by ALD (Figure 1e). At the same time, ALD-grown HfO_2_ on FG was transferred on MoS_2_ (Figure 1f). The cross-section transmission electron microscopy (TEM) images of Figure 1e,f reveal that the HfO_2_ directly grown on MoS_2_ is nonuniform, whereas the HfO_2_ transferred onto MoS_2_ via the FG transfer method is uniform and flat, maintaining a clean interface with underlying MoS_2_. We also demonstrated the FG transfer of various oxide films, including Al_2_O_3_, HfO_2_, Al-doped HfO_2_ (HAO), Hf_0.5_Zr_0.5_O_2_ (HZO), ZnO, and ZrO_2_ in Figure S2. Hereafter, the directly grown and transferred oxide films are denoted as d- and t-oxide, respectively.

To measure the breakdown voltage of the t-Al_2_O_3_ (11 nm-thick), we prepared the Au/t-Al_2_O_3_/Au device as shown in Figure 2a. Figure 2b shows that the breakdown strength of the t-Al_2_O_3_ was ∼8.82 MV cm^–1^, with a low leakage current of <10^–4^ A cm^–2^ before the breakdown, well below the international roadmap for devices and systems (IRDS) requirement for low-power devices (∼10^–2^ A cm^–2^, inset of Figure 2b). To assess dielectric properties of the t-Al_2_O_3_, we fabricated a dual-gate MoS_2_ transistor featuring a top dielectric layer of t-Al_2_O_3_ (9.5 nm-thick) and a bottom dielectric layer of 285 nm-thick SiO_2_ with Gr electrodes (see Figure 2c,d for the schematic diagram and optical microscopic image, respectively, and Methods for the device fabrication process). Figure 2e shows transfer curves (*I*_DS_–*V*_TG_) of the dual-gate MoS_2_ transistor at varying bottom-gate voltage (*V*_BG_), where the heavily doped Si was used as a bottom gate. By changing two gate voltages, the threshold voltage shift (Δ*V*_th_) was measured as a function of *V*_BG_ in the inset of Figure 2e. We calculated the relative permittivity of the t-Al_2_O_3_ (*k* = 8.3) using the linear relation between Δ*V*_th_ and *V*_BG_ (see Supporting Information 1 for the detailed calculation procedure). The obtained value aligns closely with the reported values for ALD-Al_2_O_3_. We also deposited a 14 nm-thick ZnO film on FG by ALD and transferred it onto a SiO_2_ substrate to fabricate a bottom-gate t-ZnO transistor (see Figure 2f,g for the schematic diagram and optical microscopic image, respectively, and Methods for the device fabrication process). The t-ZnO transistor in Figure 2h exhibited a high on/off ratio of >10^6^ and field-effect mobility of ∼2.93 cm^2^ V^–1^ s^–1^, comparable to ALD-ZnO devices.^28^

**Figure 2:**
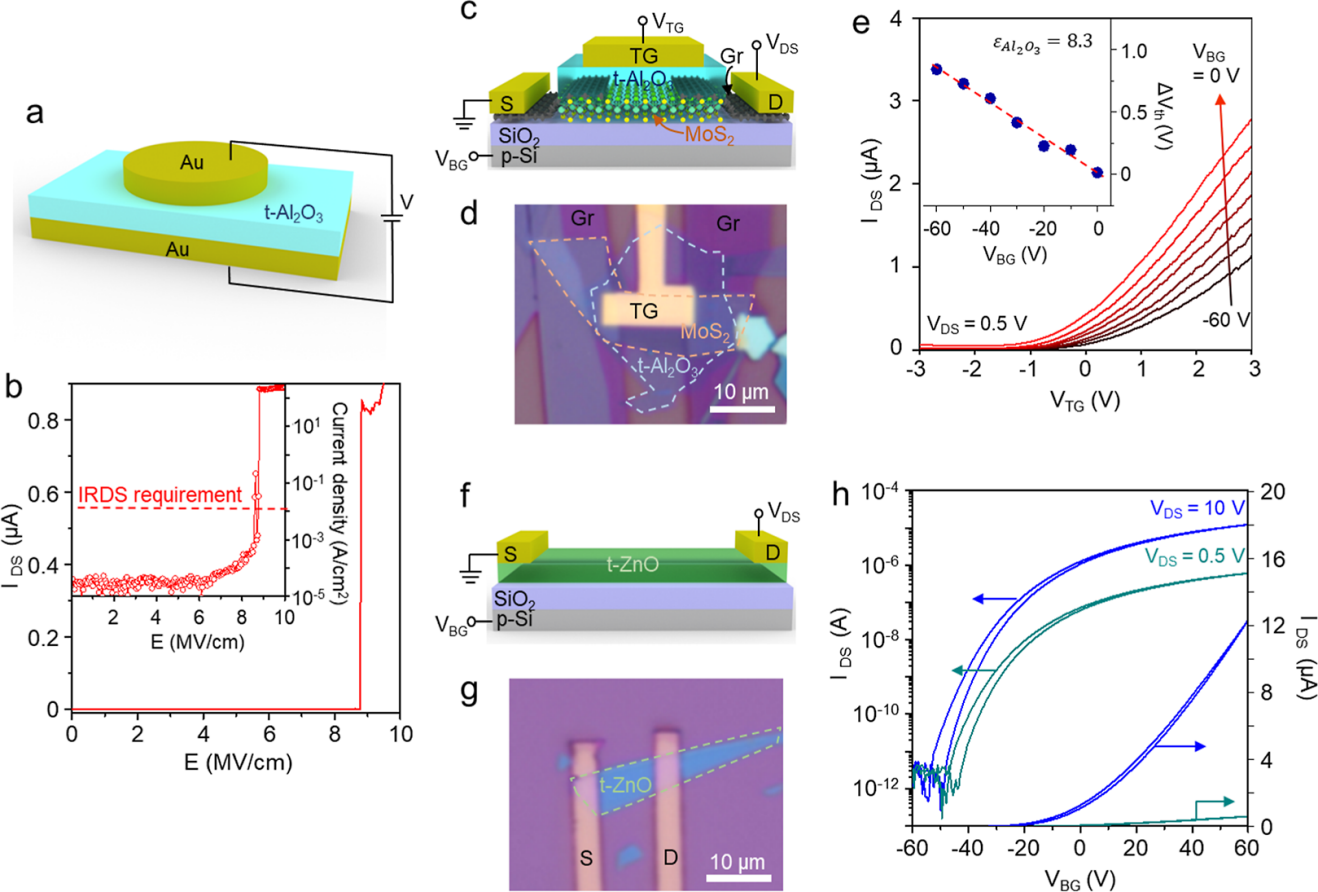
Electrical characterization of t-oxide layers. (a) Schematic of the Au/t-Al_2_O_3_/Au device for electrical breakdown measurement. (b) Breakdown current measurement of t-Al_2_O_3_ (9.5 nm-thick). Inset: leakage current density of t-Al_2_O_3_. (c) Schematic of a dual-gate MoS_2_ FET with t-Al_2_O_3_ and SiO_2_ as the top- and bottom-gate dielectrics, respectively. (d) Optical microscopic image of the fabricated dual-gate MoS_2_ FET for dual-gate measurement. (e) Transfer curves (*I*_DS_–*V*_TG_) of the dual-gate MoS_2_ FET with a sweeping top gate at varying bottom-gate voltages. Inset: threshold voltage difference (Δ*V*_th_) measured at different bottom-gate voltage. (f) Schematic of t-ZnO FET on a SiO_2_ substrate. (g) Optical microscopic image of the fabricated t-ZnO FET. (h) Transfer curves (*I*_DS_–*V*_G_) of the t-ZnO FET at *V*_DS_ = 0.5 and 10 V.

By using the FG transfer technique, ALD-grown oxide layers can be multistacked regardless of their crystal structures and lattice parameters, as illustrated in Figure 3a. The interfaces between the t-oxide layers form vdW gaps with no accumulation of interfacial strain, effectively overcoming the limitations of conventional epitaxial growth. To demonstrate this capability, we sequentially stacked t-oxide layers into a multistack heterostructure of Al_2_O_3_/ZrO_2_/HfO_2_/ZrO_2_/HfO_2_/Al_2_O_3_ on a SiO_2_ substrate using the FG transfer method. The cross-section TEM image of Figure 3b reveals uniform and ultraflat vdW interfaces between the t-oxide layers. The magnified scanning TEM (STEM) image of Figure 3c and the energy-dispersive X-ray spectroscopy (EDS) maps of Figure 3d (obtained from the red-dashed box in Figure 3b) confirm the formation of clean vdW gaps with no elemental intermixing. These results indicate that uniform ALD-oxide layers on FG can be assembled into vdW heterostructures without being constrained by lattice mismatches.

**Figure 3:**
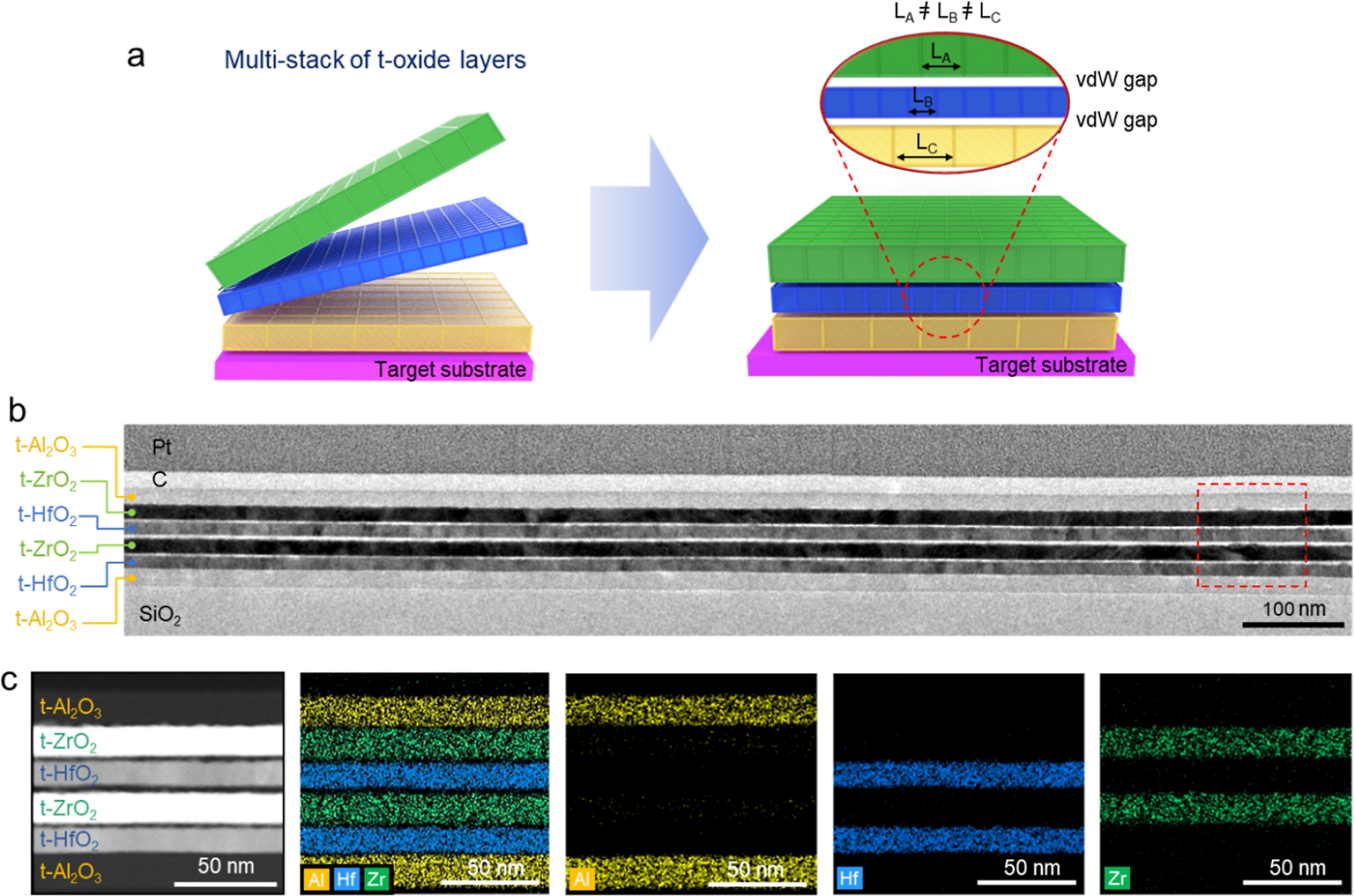
Fabrication of the multistacked heterostructure of ALD-oxides by the FG transfer method. (a) Schematic illustration of the multistacking process of various oxide layers with different crystal structures and lattice parameters. There is no constraint between transferred oxide layers due to the absence of chemical bonds and presence of vdW gaps. (b) Cross-section TEM image of Al_2_O_3_/ZrO_2_/HfO_2_/ZrO_2_/HfO_2_/Al_2_O_3_, multistacked heterostructure of ALD-grown oxide layers. (c) STEM image of the stack obtained from the red-dashed box in (b). EDS mapping image of combined Al/Hf/Zr and individual Al, Hf, and Zr elements.

To verify the thermal stability of the vdW interface between t-oxide layers, we prepared two samples: d-Al_2_O_3_/t-ZnO and t-Al_2_O_3_/t-ZnO, as shown in Figure 4a–d. The cross-section STEM image of Figure 4a and EDS line profiles of Figure 4b (obtained from the red-dashed box in Figure 4a) confirmed that the d-Al_2_O_3_/t-ZnO sample had a rough and uneven interface with an intermixed region (∼3.8 nm-thick) of Al and Zn. In contrast, a similar analysis in Figure 4c,d (EDS profile was obtained from the blue-dashed box in Figure 4c) indicated that the t-Al_2_O_3_/t-ZnO sample exhibited a uniform and clean vdW interface with no intermixed region. Notably, intermixing at the interface, frequently observed after postannealing, was effectively suppressed in the transferred oxide layers (Figure S5). After postannealing at 400 °C for 3 h, the d-Al_2_O_3_/t-ZnO interface became blurred, indicating further intermixing. Meanwhile, no intermixing was observed at the t-Al_2_O_3_/t-ZnO interface after identical annealing. These findings confirm that the FG transfer technique effectively mitigates intermixing issues during postannealing and addresses thermal budget challenges in multilayered structures.^29^

**Figure 4:**
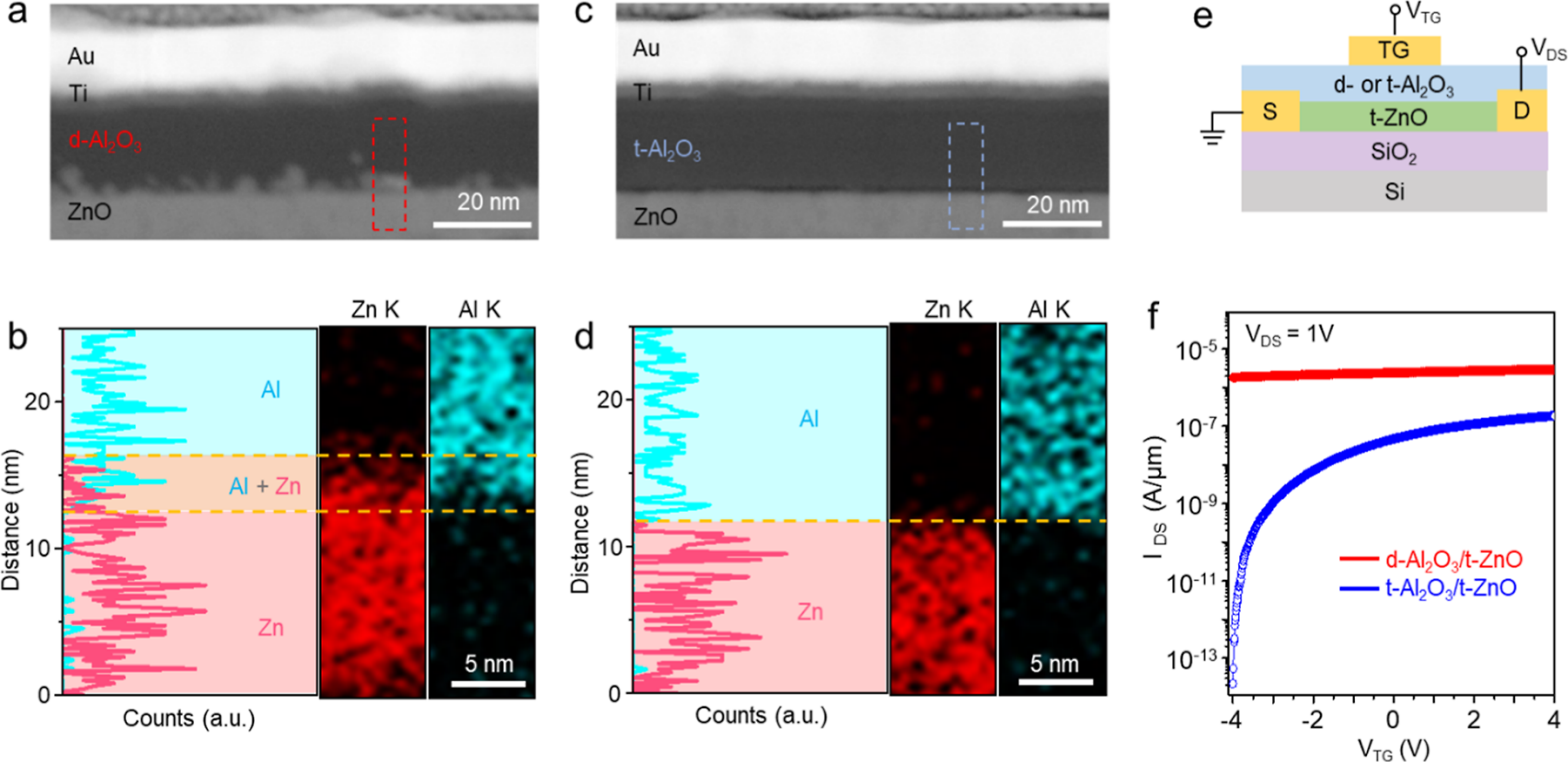
Interfacial stabilities of d-Al_2_O_3_/ZnO and t-Al_2_O_3_/ZnO. (a) Cross-section STEM of d-Al_2_O_3_/ZnO. (b) EDS line profile and mapping images of Zn and Al in d-Al_2_O_3_/ZnO (obtained from the red-dashed box in (a)). (c) Cross-section STEM of t-Al_2_O_3_/ZnO. (d) EDS line scan and EDS mapping image with Zn and Al of t-Al_2_O_3_/ZnO (obtained from the blue-dashed box in (c)). (e) Schematic device geometry of the fabricated ZnO FET. (f) Transfer curves (*I*_DS_–*V*_TG_) of d-Al_2_O_3_/ZnO and t-Al_2_O_3_/ZnO FETs.

To evaluate the electrical properties of the interfaces, we fabricated top-gate ZnO devices with d- and t-Al_2_O_3_ dielectric layers on the 285 nm-thick SiO_2_/Si, as shown in Figures 4e and S3. The transfer curves in Figure 4f reveal that the t-Al_2_O_3_/t-ZnO device achieved a high on/off current ratio of ∼10^7^, whereas the d-Al_2_O_3_/t-ZnO device exhibited high conductivity with negligible gate tunability. Furthermore, the transfer curves of t-Al_2_O_3_/t-ZnO, d-Al_2_O_3_/t-ZnO, and t-ZnO devices (Figure S4), operated using a *V*_BG_, clearly demonstrate that the direct deposition of Al_2_O_3_ on the ZnO channel induces significant n-doping, resulting in increased off current. Notably, the t-Al_2_O_3_/t-ZnO device displayed negligible changes in its transfer curves compared with t-ZnO with no capping layer. This observation suggests that the t-Al_2_O_3_ layer has a minimal impact on the ZnO channel, likely due to the presence of the vdW gap and the absence of intermixing.

We extended the FG transfer to the transfer of patterned oxide layers, as depicted in Figure 5a. First, CVD-grown monolayer Gr on a Cu foil film was transferred onto a SiO_2_ substrate, followed by patterning it into a desired shape through e-beam lithography and oxygen plasma etching. After fluorination of Gr, the oxide layer was deposited by ALD. By applying PC film on the patterned oxide layer/FG and then detaching it, only the oxide layer deposited on FG was selectively delaminated along with the FG from the substrate. The FG can either be etched away or left intact, depending on the intended application. Finally, the patterned oxide layer was transferred onto a target substrate, and the PC film was removed (see Methods for the detailed transfer process). Figure 5b shows the patterned Gr and transferred Al_2_O_3_ patterns with star, cross, square, and triangle shapes.

**Figure 5:**
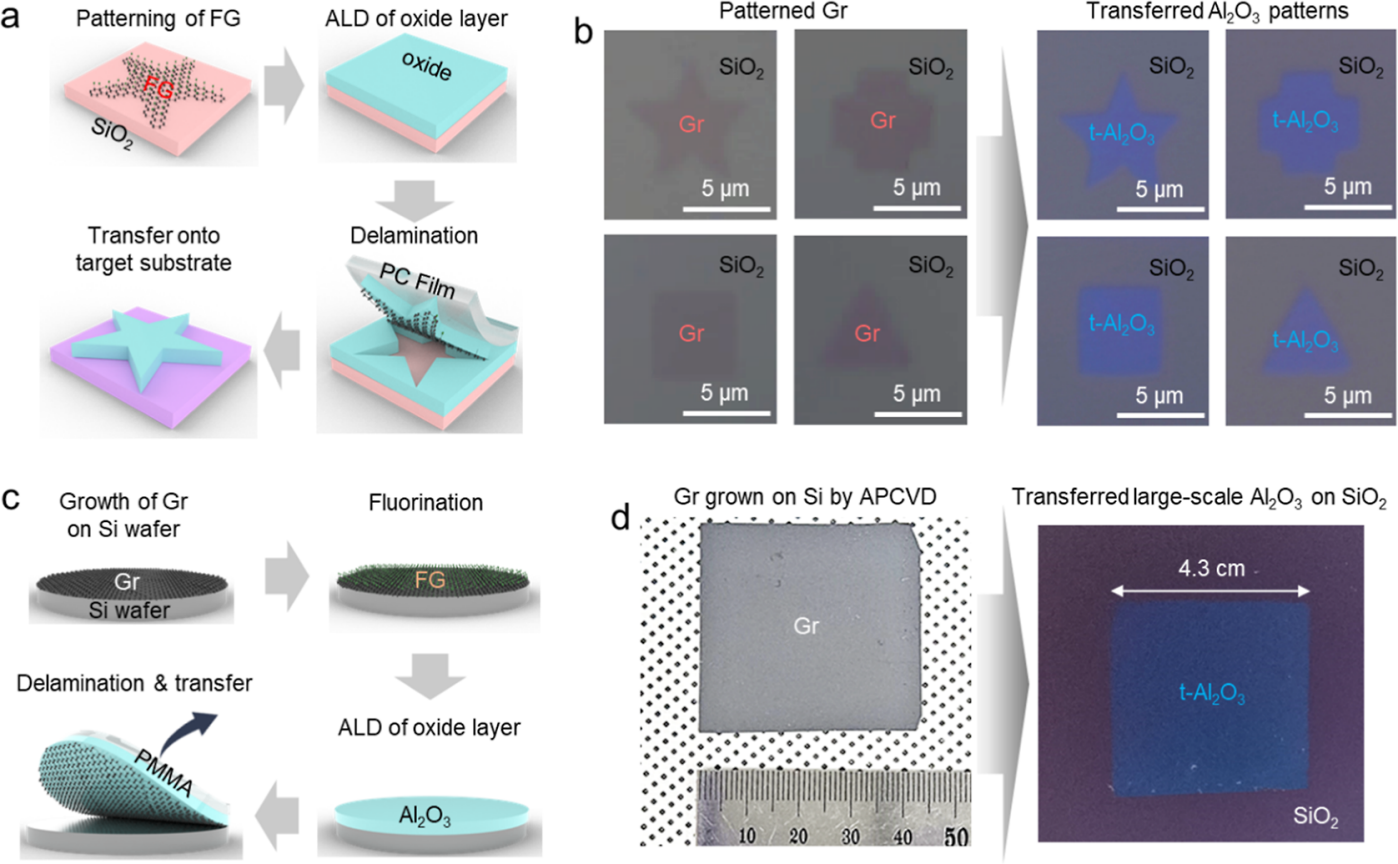
Transfer of patterned ALD-grown oxide layers by the FG transfer method. (a) Schematic FG transfer process of patterned ALD-grown oxide layers. (b) Optical microscopic images of patterned Gr and transferred Al_2_O_3_ with star, cross, square, and triangle shapes. (c) Schematic FG transfer process of large-scale ALD-grown oxide layers using directly grown Gr on a Si wafer. (d) Optical microscopic images of APCVD-grown Gr and transferred Al_2_O_3_ on a SiO_2_ substrate.

Additionally, we demonstrated the scalability of the FG transfer using large-scale Gr directly grown on a Si wafer. As depicted in Figure 5c, multilayer Gr was grown on the Si wafer using atmospheric pressure CVD (APCVD) as described in our previous report (see Methods for the APCVD process).^30^ After fluorination of Gr, the oxide layer was grown by ALD. Then, poly methyl methacrylate (PMMA) was spin-coated, and thermal release tape (TRT) was attached, as illustrated in Figure S1c.^31^ Finally, the large-scale oxide layer is delaminated along with FG and transferred onto a target substrate (see the Methods for the detailed transfer process). Figure 5d shows multilayer Gr grown directly on a Si wafer by APCVD and a transferred large-scale Al_2_O_3_ layer with a length of 4.3 cm on a SiO_2_ substrate. These results show the great potential of the FG transfer technique for stacking patterned oxide layers on a large scale.

## Conclusion

In conclusion, we introduce the FG transfer technique as an innovative solution for integrating ALD-grown oxide films, addressing critical challenges in M3D integration such as lattice mismatch, interlayer diffusion, and material intermixing. This technique enables the fabrication of multilayered oxide films with clean vdW interfaces, free from intermixing or interfacial strain. The FG transfer process maintains the structural and electrical integrity of the oxide films, even after high-temperature postannealing, effectively overcoming thermal budget constraints. Furthermore, the scalability and versatility of this approach are demonstrated through the successful integration of patterned and large-area ALD-oxide films, including inch-scale layers and multistack heterostructures with complex compositions. These results highlight the capability of the FG transfer technique to overcome the limitations of conventional epitaxial growth methods, offering a pathway to high-performance device fabrication and industrial-scale applications. Overall, the FG transfer technique provides a reliable and scalable platform for next-generation semiconductor manufacturing, facilitating advanced M3D integration and opening new possibilities for the design of vdW heterostructures.

## Methods

### FG Transfer Process

First, Gr was mechanically exfoliated on a SiO_2_/Si substrate. For the fluorination process, Gr was exposed to XeF_2_ gas under a pressure of 400 Pa for 5 s, repeated over 60 cycles, using a Samco VPE-4F system.^17–22^ After the ALD growth of oxide layers, the samples were annealed on a hot plate at 400 °C for 4 h to reduce the adhesion of the oxide films. Finally, we prepared a PC/polydimethylsiloxane (PDMS) lens for the pick-up transfer of the oxides.^23^ By controlling how this lens contacts the target sample (Figure S2), we can selectively transfer only the oxides or both the oxides and the underlying Gr. When the PC/PDMS lens contacts only a portion of the target sample, it selectively transfers the oxide layer from the contacted area, whereas full contact with the entire sample enables transfer along with the underlying Gr. Notably, the substrate temperature during the detachment of the target material is set to 140 °C. Finally, the PC film is etched away by a chloroform etchant. For large-scale FG transfer, Gr was directly grown on a Si substrate using APCVD at atmospheric pressure and 1020 °C with a flow of CH_4_ (25 sccm), H_2_ (20 sccm), and Ar (80 sccm) for 2 h.^30^ After XeF_2_ treatment and the ALD process, PMMA was spin-coated and TRT was attached instead of the PC/PDMS lens. Owing to the weak adhesion between directly grown Gr and the Si substrate, the Gr was detached from the substrate in the FG transfer process. So, to remove the Gr, we used mild oxygen plasma treatment using PLASOL PS-150 (60 W/100 kHz/10 s).

### ALD Growth of Oxide Layers

Various oxide thin films were grown on the FG at 200 °C by ALD. In the ALD process, trimethylaluminum (TMA), tetrakis­(ethylmethylamido)hafnium (TEMAH), diethylzinc (DEZ), and tetrakis­(ethylmethylamido)zirconium (TEMAZ) were used as precursors for Al_2_O_3_, HfO_2_, ZnO, and ZrO_2_, along with H_2_O for all processes.

### FG Transfer of Patterned Oxide Layers

To transfer patterned ALD-oxide thin films, CVD-Gr grown on Cu foil was transferred to a SiO_2_ substrate by a wet transfer method.^32^ E-beam lithography was performed to pattern the Gr into specific shapes such as a star, cross, square, and triangle. The XeF_2_ treatment and ALD process were carried out, and then the pick-up process was used to transfer the patterned oxide layers with a PC/PDMS lens. Note that the underlying Gr is also transferred during this process. Therefore, to transfer only the ALD-oxide without the Gr, the patterned heterostructure was detached, then flipped over, and subjected to mild oxygen plasma treatment to remove the Gr.

### Structural Characterizations

AFM images were obtained in noncontact mode using NX-10 (Park Systems). Raman spectroscopy with a 532 nm laser (Jasco, NRS-4500) was used. HR-TEM and STEM images were captured by using aberration-corrected TEM (JEOL JEM-ARM 200F) at an operating voltage of 80 kV. A focused ion beam (FIB) system (Helios G4, Thermo Fisher Scientific, USA) was used to prepare the samples for cross-sectional TEM imaging.

### Device Fabrication and Electrical Measurements

Stacks of ALD-grown oxide layers or Gr electrodes were fabricated by the pick-up technique on a target substrate. For t-Al_2_O_3_/MoS_2_ FET, ALD-grown Al_2_O_3_ on FG and Gr electrodes were stacked by the pick-up technique on the MoS_2_ channel. E-beam lithography was performed to develop patterns of the metal pads. Metals of Au/Ti (40/10 nm) were then deposited on the exposed Gr electrodes by using an e-beam evaporator. For the t-Al_2_O_3_/t-ZnO FET, ALD-grown Al_2_O_3_ on FG was stacked onto t-ZnO, where Au/Ti (15/10 nm) metal electrodes were prepatterned using the e-beam lithography and an e-beam evaporator. Similarly, the top-gate electrode (Au–Ti) was also formed on the t-Al_2_O_3_ layer of both FETs. The electrical measurements of the devices were conducted using a parameter analyzer (Keithley 4200A) at room temperature under vacuum conditions.

## Supplementary Material



## Notes

Dual-gate method; schematic illustration of the FG transfer processes for transfer of ALD-grown oxide layers and FG; FG transfer of various oxide layers; comparison of d- and t-Al_2_O_3_ in ZnO FETs; effect of d- and t-Al_2_O_3_ on ZnO FETs; and interfacial characteristics of annealed d-Al_2_O_3_/ZnO and t-Al_2_O_3_/ZnO (PDF)
