# Supplementary material for: Vertical Stacking of Atomic-Layer-Deposited Oxide Layers via a Fluorinated Graphene Transfer Technique
Source: ACS Nano. 2025 Jun 13;19(25):23186–92. doi: 10.1021/acsnano.5c04669 (PMC12224289; doi:10.1021/acsnano.5c04669)
Supplement: Supplementary file 1 [file nn5c04669_si_001.pdf]

Supporting Information

Vertical Stacking of Atomic-layer-deposited Oxide Layers via Fluorinated Graphene Transfer Technique

AUTHOR NAMES

*Hyunjun Kim[1], Huije Ryu[1], Hyun Woo Jeong[1], Jiwoo Kim[1], Donghoon Moon[1], Sahngik Aaron Mun[1], Cheol Seong Hwang[1], Min Hyuk Park[1], Jangyup Son[2,3,4], Gwan-Hyoung Lee[1]**

AUTHOR ADDRESS

[1] Department of Materials Science and Engineering, Seoul National University, Seoul 08826, Republic of Korea

[2] Functional Composite Materials Research Center, Korea Institute of Science and Technology (KIST), Jeonbuk 55324, Republic of Korea

[3] Department of JBNU-KIST Industry-Academia Convergence Research, Jeonbuk National University, Jeonju 54896, Republic of Korea

[4] Nanoscience and Technology, KIST School, University of Science and Technology, Seoul 02792, Republic of Korea.

Supplementary Note 1:

Measurement of dielectric constant in a dual-gate MoS_2 device

A shift in the transfer curve (I_{DS}-V_{TG}) was observed at the applied bottom gate voltage, and plotting the shift in threshold voltage as a function of bottom gate voltage yields a linear relation, as shown in the inset of Fig. 2e. The total charge in the dual-gate MoS_2 device can be described by Eq. (1):

$$Q_{total} = C_{Top}V_{TG} + C_{Bottom}V_{BG} \quad (1)$$

At the threshold voltage, the net-induced charge becomes zero. Therefore, the variation in the threshold voltage of the top gate can be expressed as follows:

$$\Delta V_{TG,th} = -\frac{C_{Bottom}}{C_{Top}}\Delta V_{BG} \quad (2)$$

Capacitance (C) is related to the dielectric constant (ε), area (A), and thickness (d) of the dielectric material, and since $C = \frac{\varepsilon A}{d}$, the ratio of capacitances can be written as:

$$\frac{C_{Bottom}}{C_{Top}} = \frac{\varepsilon_{Bottom}d_{Top}}{\varepsilon_{Top}d_{Bottom}} \quad (3)$$

As shown in Fig. 4f, the slope of $\Delta V_{TG,th}$ for ΔV_{BG} is -0.015664, which is $-\frac{C_{Bottom}}{C_{Top}}$. For the given values of ε_{Bottom} (= 3.9), d_{Top} (= 9.5 nm), and d_{Bottom} (= 285 nm), the dielectric constant of Al_2O_3 (ε_{Top}) is 8.3.

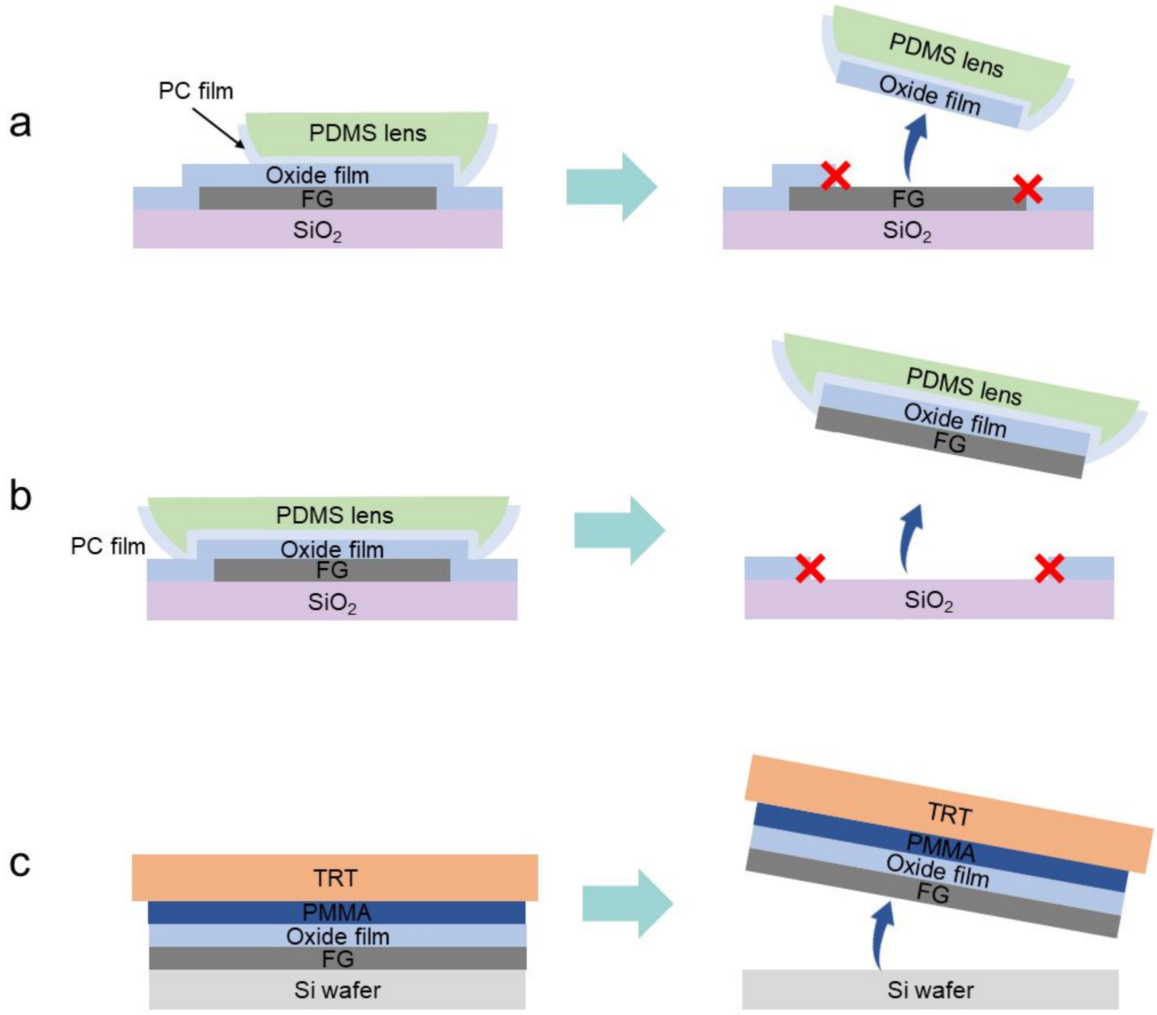

Figure S1. Schematic illustration of the FG transfer processes for transfer of ALD-grown oxide layers and FG. a) Transfer process for selectively detaching only the ALD-grown oxide layer from the FG. When PC/PDMS makes partial contact with the FG area, only the oxide layer is removed from the contacted region. b) Transfer process for detaching the FG and oxide layer together. When PC/PDMS fully contacts the FG area, the entire FG/oxide heterostructure is detached. c) Transfer process for large-scale oxide layer. To transfer the large-scale oxide layer deposited on the FG (fluorinated APCVD-grown Gr on a Si wafer), PMMA was spin-coated, and FG/oxide was detached using TRT. The whole FG/oxide heterostructure was detached

from the Si wafer. Before its transfer onto a target substrate, FG was removed by oxygen plasma.

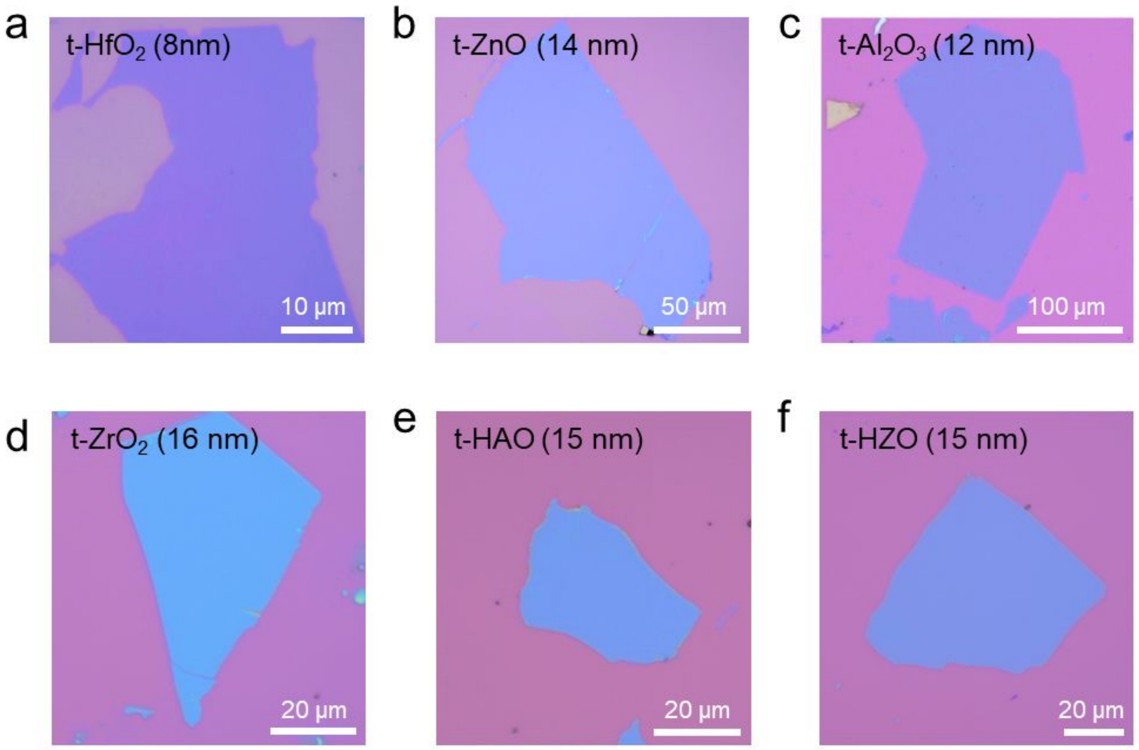

Figure S2. FG transfer of various oxide layers. Optical microscopi c images of a) HfO_2, b) ZnO, c) Al_2O_3, d) ZrO_2, e) HAO, and f) HZO. The thicknesses of oxide layers are indicated in the images.

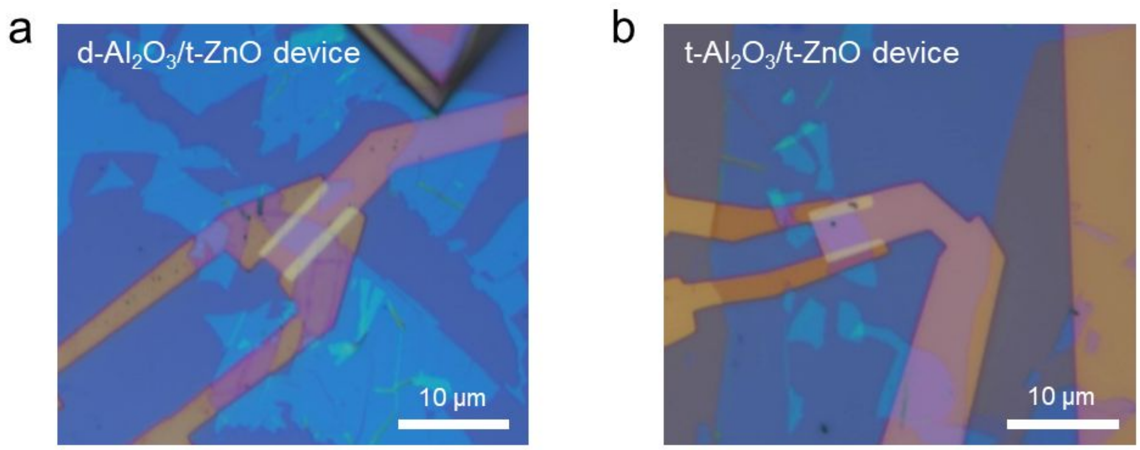

Figure S3. Comparison of d- and t-Al_2O_3 in ZnO FETs. Optical microscopic images of a) d-Al_2O_3/ZnO FET and b) t-Al_2O_3/ZnO FET.

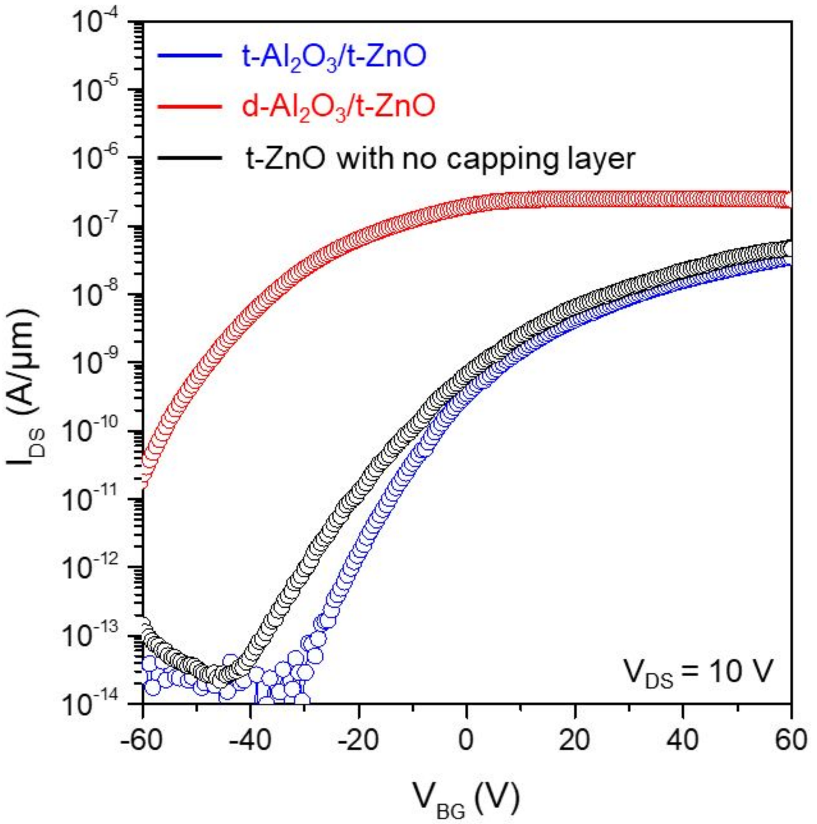

Figure S4. Effect of d- and t-Al_2O_3 on ZnO FETs. Transfer curves (I_{DS}-V_{BG}) of d-Al_2O_3/ZnO, t-Al_2O_3/ZnO, and ZnO (no capping layer) FETs.

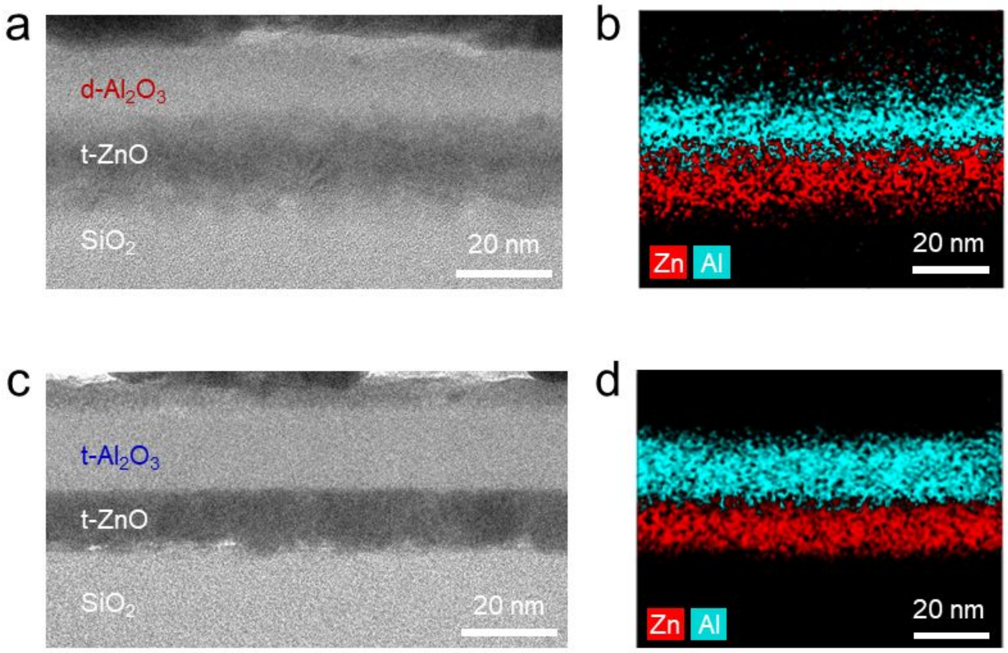

Figure S5. Interfacial characteristics of annealed d-Al_2O_3/ZnO and t-Al_2O_3/ZnO. a) Cross-section STEM and b) EDS mapping images of d-Al_2O_3/ZnO after annealing at 400 ^{o}C for 3h. c) Cross-section STEM and d) EDS mapping images of t-Al_2O_3/ZnO after annealing at 400 ^{o}C for 3h.
